# Development of energy intensive multifunction cavitation technology and its application to the surface modification of the Ni-based columnar crystal superalloy CM186LC

**DOI:** 10.1016/j.heliyon.2021.e08572

**Published:** 2021-12-08

**Authors:** Toshihiko Yoshimura, Yuji Sugae, Takayuki Ogi, Fumihiro Kato, Masataka Ijiri

**Affiliations:** a Department of Mechanical Engineering, Sanyo-Onoda City University, 1-1-1 Daigaku-dori, Sanyo-Onoda, Yamaguchi, 756-0884, Japan; b Department of Mechanical Systems Engineering, Tokyo Metropolitan University, 1-1 Minami-Osawa, Hachioji, Tokyo, 192-0397, Japan

**Keywords:** Multifunction cavitation, Water jet cavitation, High-temperature high-pressure cavitation, Ni-based superalloy, Rafting, Energy-intensive multifunction cavitation

## Abstract

The present work demonstrates a technique for the hot forging of metal surfaces in water at 1000 °C or higher, termed energy-intensive multifunctional cavitation (EI-MFC). In this process, the energy of cavitation bubbles is maximized, following which these bubbles collide with the metal surface. This technique will be employed to improve the surface structure of CM186LC/DS, a Ni-based columnar crystalline superalloy used to manufacture the rotor blades of jet engines and gas turbines that are exposed to high-temperature oxidizing environments, with the aim of improving creep strength. EI-MFC processing induces compressive residual stress in the metal that prevents the occurrence of surface cracks and also increases surface hardness, improves corrosion resistance, and increases the coefficient of friction. The latter effect can enhance the adhesion of thermal barrier coatings applied to Ni-based superalloys by thermal spraying. The technology demonstrated herein can be applied to present-day jet engine and gas turbine components and also to the production of hydrogen combustion turbines operating at 1700 °C with higher combustion efficiency than the current 1500 °C class gas turbines. In addition, the high processing energy obtained using the EI-MFC technique has the potential to flatten rough surfaces resulting from the stacking pitches of various metals manufactured using three-dimensional printers, and so improve surface strength.

## 1 Introduction

Ni-based superalloys can be classified as either cast or forged alloys, and these metals have applications to different jet engine components. As an example, Ni-based superalloys are used to fabricate high-pressure turbine blades employed in combustion chambers as well as the disks that support these blades. These materials are also used to produce low-pressure turbine blades that approach exhaust gases. These alloys are able to withstand high temperatures and pressures and; in fact, the temperature limit of the Ni-based alloy from which turbine blades are fabricated determines the temperature of the combustion chamber and is directly linked to the efficiency of the engine and gas turbine. Therefore, the development of alloys with higher temperature limits is an important aspect of designing new high-efficiency engines and turbines.

To date, research regarding Ni-based superalloys has been based on finding new alloys as well as microstructure control and related processing technologies. Up to the 1950s, these materials were wrought alloys, while unidirectionally solidified conventional casting (CC) alloys were produced in the 1960s (Reed, 2006). This new process eliminated crystal grain boundaries perpendicular to the longitudinal direction of the turbine blades, and led to the development of directional solidification (DS) alloys. Since then, single-crystal alloys that do not contain grain boundaries (which tend to limit high temperature performance) have been put into practical application and are currently installed in many aircraft engines. Single crystals are known to exhibit greater rupture resistance (that is, longer creep lifetimes) at high temperatures and constant stress (Reed, 2006; Pollock and Tin, 2006). As an example, Ni-based superalloys retain their strength up to 800 °C as a result of reinforcement with a γ′ Ni_3_Al phase and exhibit high strength even at 1000 °C. This high temperature performance is one of the primary reasons for the use of these metals. Even so, the volume-based proportion of the γ′ phase in these materials is not necessarily high, and the creep resistance is maximized at a proportion of approximately 70%. Therefore, it is necessary to control the volume proportion of the γ′ phase in such alloys.

Technologies that utilize high-pressure water jets have been developed for many years now, and this technique has been applied in the fields of cleaning, coal and rock mining, demolition, industrial machining and impulse fragmenting (Summers, 1995). Water jetting can be enhanced by using polymeric additives, cavitation bubble collapse, abrasive injection and hydromechanical cutting (Summers, 1995). In this research, the basic technology is based on the collapse of cavitation bubbles. As an example, Kim et al. (2005) and Gao and Wu (2011) used shot peening associated with the mechanical collisions of particles to apply compressive residual stress to metal surfaces. Hashish (1984, 1991) also developed an abrasive water jetting technique in which an abrasive was added to a 300 MPa jet. Kling (1970) and Summers et al. (1987) devised a process known as water jet cavitation (WJC) that took advantage of the cavitation collapse phenomenon occurring on the surface of a material when applying a 1000 MPa jet. The application of WJC to nuclear power generation was studied beginning in 1990. WJC has also been developed as a peening technique to change the residual stress of welded parts from tensile to compressive as a preventative maintenance technique (Saitou et al., 2003). However, shot peening techniques have also been used to apply residual compressive stress to various surfaces. Yoshimura et al. (2016) developed a processing technology known as multifunction cavitation (MFC) that provides the advantages of both water jetting and ultrasonic cavitation, and has applications in cleaning, biotechnology and chemistry. MFC imparts residual compressive stress to various metals and also causes structural changes, such as in Cr–Mo steel specimens that exhibit the formation of a toughened layer without voids or cracks just below the surface. An ultra-high-temperature and high-pressure cavitation (UTPC) method has also been reported by Yoshimura et al. (2018), based on a swirl nozzle attached to a water jet nozzle. The photons emitted from cavitation bubbles during MFC and UTPC was quantified and also determined the bubble temperatures.

In the present research, a technology termed energy intensive MFC (EI-MFC), which is an iteration of the UTPC method providing increased cavitation energy, was developed. The viability of improving the properties of the Ni-based columnar crystal superalloy CM186LC (which is used in extremely high-temperature corrosive environments) by employing this new process was examined. This work also examined whether or not structural changes occurring in Ni-based superalloys used in jet engines and gas turbine blades heated to approximately 1300 °C could be reproduced in water in conjunction with this new technique.

## 2 Material and methods

**Figure 1:**
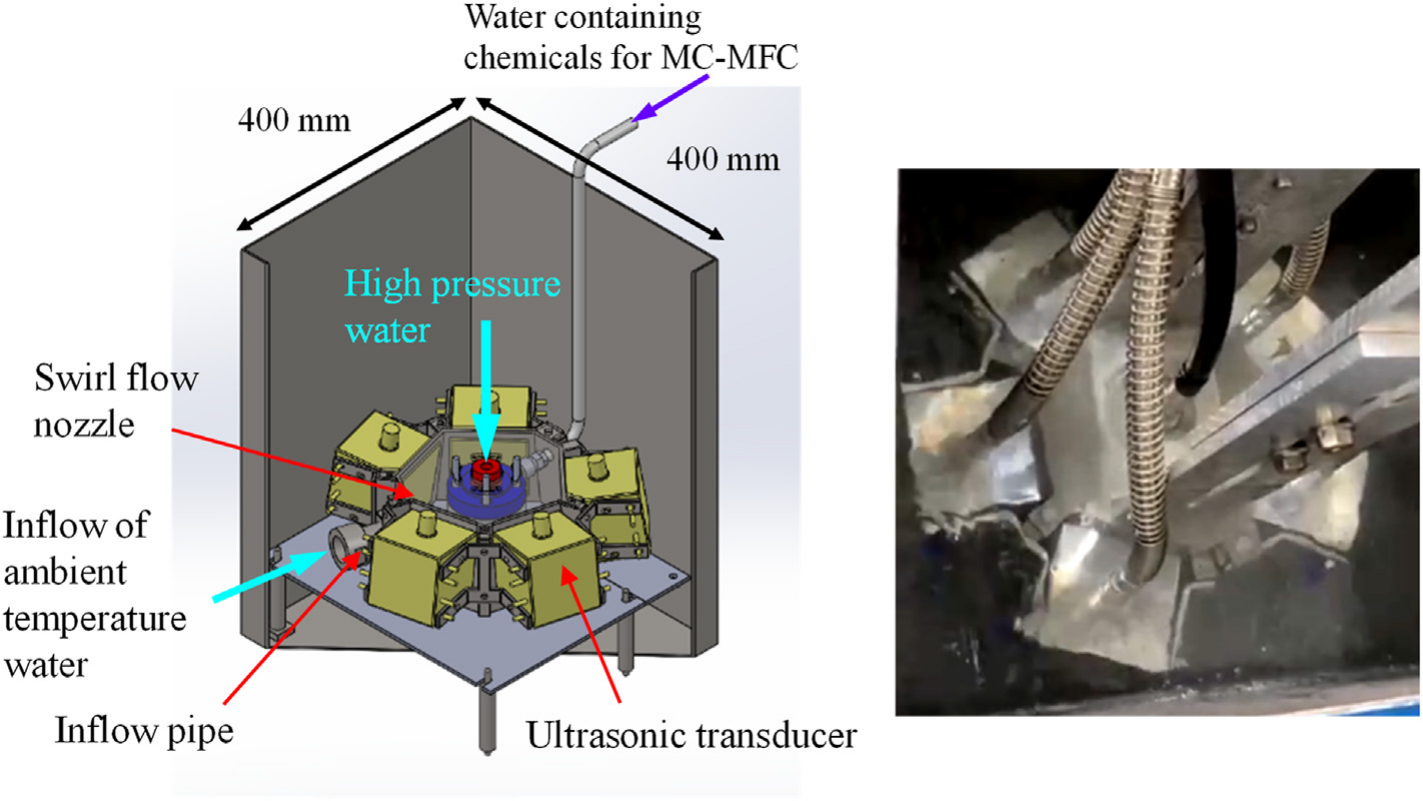
The energy-intensive multifunction cavitation apparatus.

Figure 1 shows a three dimensional (3D) computer aided design diagram and a photographic image of the newly developed EI-MFC equipment. In this apparatus, five 40 W ultrasonic transducers (WSC28, Honda Electronics Co., Ltd.) operating at 28 kHz are arranged in a pentagonal shape. This odd numbered arrangement was used to avoid the possibility that the sound pressure generated by the ultrasonic waves could be canceled when using an even-numbered shape such as a hexagon. Similar to the UTPC process, water at ambient temperature enters from an inflow hole as the dynamic pressure increases and the static pressure decreases due to the operation of the WJ, and a swirling flow is generated. As a result of these effects, the pressure around the WJ jet in the swirl nozzle is reduced and the number of bubbles increases as the bubble size increases and the cavitation number, Ca, decreases. The temperature and pressure inside the bubbles are maximized by increasing the size of the initial bubbles as well as by ensuring that the bubbles undergo isothermal expansion and adiabatic compression (Rayleigh, 1917; Plesset, 1949) via the application of ultrasonic irradiation. In addition, by reducing the distance between the swirling nozzle and the specimen table, it takes time to flow out to the outside, because flow rate from the gap between the swirling nozzle and the specimen table decreases. Consequently, the number of isothermal expansions and adiabatic compressions is increased and the bubble energy becomes extremely high. This device can also function so that it is equivalent to the mechanochemical MFC (MC-MFC) process that has been previously researched (Ijiri et al., 2021a). Specifically, in the case that the WJ nozzle is used as an ejector nozzle, atmospheric pressure can introduce various chemicals into the sidestream of the ejector nozzle (Yoshimura et al., 2014) from the pipe shown in Figure 1. This occurs as a result of the decrease in static pressure around the WJ jet and permits concentrated energy MC-MFC processing.

The most unique feature of the EI-MFC technique is that ultrasonic waves are radiated from the circumferential direction of the WJ jet. As such, the number of bubbles undergoing isothermal expansion and adiabatic compression is increased relative to the quantities associated with UTPC, in which the WJ jet is irradiated with ultrasonic waves from a single vertical direction.

**Figure 2:**
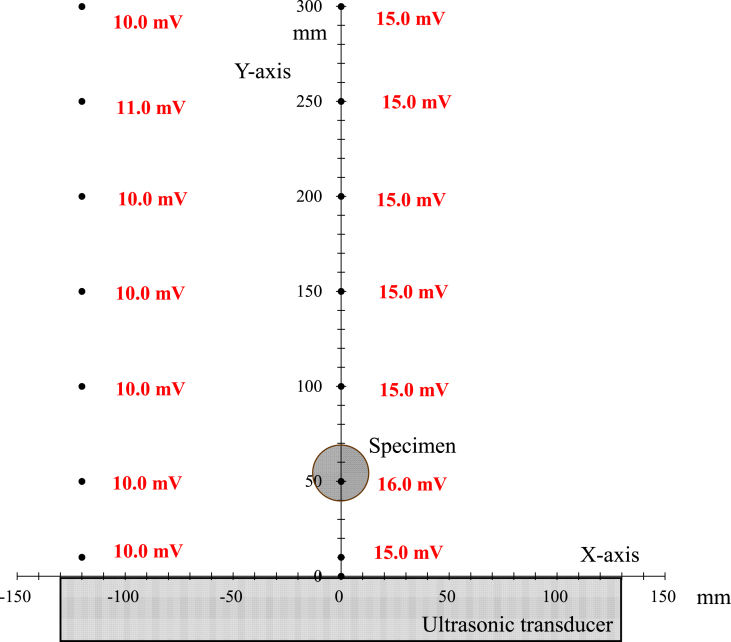
Relative sound pressures measured using a sensor at specific distances from the center of the ultrasonic transducer (Error bar at each measurement point ≒ ± 0.3 mV).

Figure 2 shows the relative sound pressures determined using a pressure sensor (HUS-3 Portable Sonic Monitor, Honda Electronics Co., Ltd.) at specific distances from the center of the ultrasonic transducer.

The absolute sound pressure in water irradiated with ultrasonic waves cannot be measured because numerous vacuum bubbles repeatedly collapse due to the cavitation phenomenon, which interferes with the measurement process. Therefore, a method was developed in which a piezoelectric element was attached to a stainless steel vibration transmission rod, such that the vibrations resulting from the cavitation shock waves in the water were received and displayed as a voltage that corresponded to the relative sound pressure. Increasing the output (that is, the wattage) of the ultrasonic transmitter was found to increase the display value (in mV) of the sound pressure gauge, confirming that these two variables were directly proportional to one another. Thus, although this method was not able to determine the absolute pressure value (in Pa), it generated a voltage proportional to the shock wave associated with cavitation. For the same reasons, relative sound pressure gauges are also used in ultrasonic cleaning processes.

As in previous research (Yoshimura et al., 2021) involving UTPC, a high output oscillator (WS-1200-28N, Honda Electronics Co., Ltd.) was employed together with an ultrasonic output of 1200 W and an ultrasonic frequency of 28 kHz, while the present EI-MFC technique employed an ultrasonic output of 40 W. In the present experiments, six WSC28 oscillators operating at a frequency of 28 kHz were arranged in five rows in the x-axis direction, with the center of the third row assigned the coordinates (0,0). The relative sound pressure was determined to be 15 mV at a position 10 mm away from the center of the ultrasonic transducer. In the case of the UTPC trials (SFN-MFC: Swirl Flow Nozzle – Multifunction Cavitation) the apparatus was equipped with a swirl nozzle and the specimen was installed at a position 54 mm (equivalent to 1 wavelength at a frequency of 28 kHz) from the center, at which point the relative sound pressure was in the range from 15 to 16 mV.

**Figure 3:**
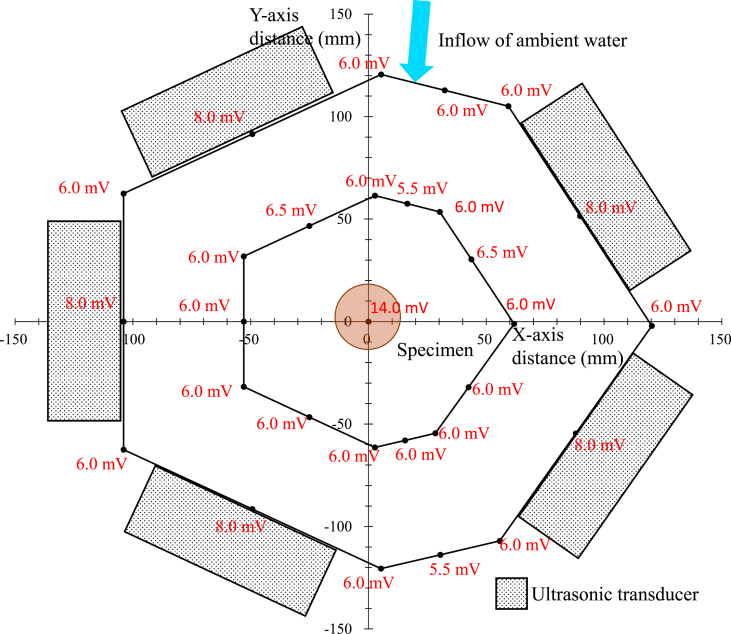
The arrangement of ultrasonic oscillators and sound pressure distribution in the energy-intensive multifunction cavitation apparatus.

Figure 3 shows the arrangement of the ultrasonic oscillators and the sound pressure distribution during the EI-MFC process. The relative sound pressure in the immediate vicinity of the oscillator was 8 mV and so was less than the value of 15 mV associated with UPTC. However, the pressure at the point at which the sample was processed by WJC was 14 mV, which was equivalent to the UPTC value.

The distribution of the WJC bubble cloud and the unsteady flow associated with this process were examined both analytically and experimentally by Peng et al. (2018), but such analyses have not been performed in the case of MFC. In addition, the vibration of the WJC bubble cloud largely depends on the porosity (that is, the volume fraction). Peng et al. (2015) reported that the oscillation frequency of this cloud is lower than the frequency with which individual bubbles move, and that the oscillation decays very slowly at high void ratios. In contrast to the UTPC process, which involves sending out ultrasonic waves from one direction, the EI-MFC apparatus shown in Figure 3 generates ultrasonic waves all around the sample so that the void ratio is decreased and the vibrational frequency of the bubble cloud increases.

**Figure 4:**
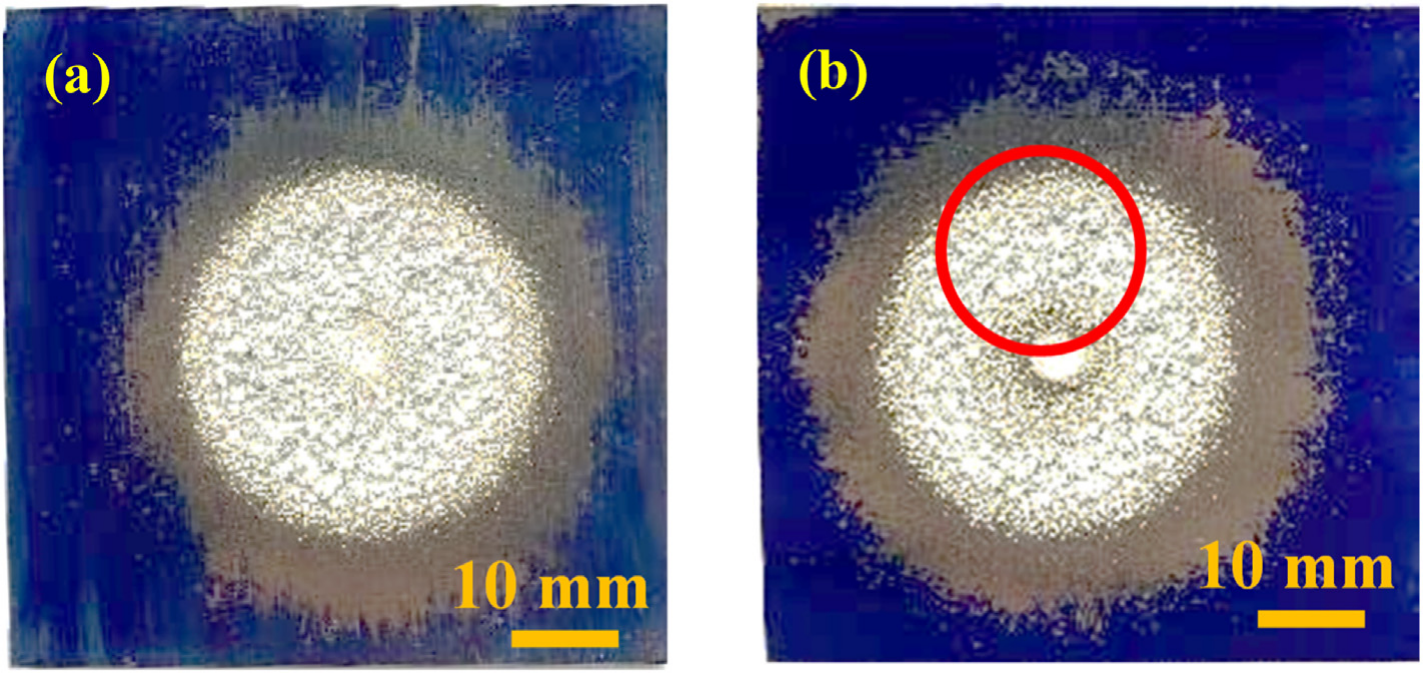
Photographic images of specimen surfaces after (a) UTPC processing for 30 min and (b) EI-MFC processing for 30 min.

Figure 4 provides photographic images of the surfaces of pure A1050 aluminum samples after processing by either UTPC or EI-MFC for 30 min. The surface of the UTPC sample was evidently processed uniformly, while the surface of the EI-MFC specimen was heavily processed on the area shown by the circle. This difference is attributed to the variations in the two different apparatuses. Specifically, the UTPC swirl nozzle was circular and had two inflow holes, while the EI-MFC unit was pentagonal and had one inflow hole. Even so, both specimens exhibited approximately the same mass loss, presumably because the EI-MFC technique provided a higher degree of processing in the localized area subjected to peening.

**Figure 5:**
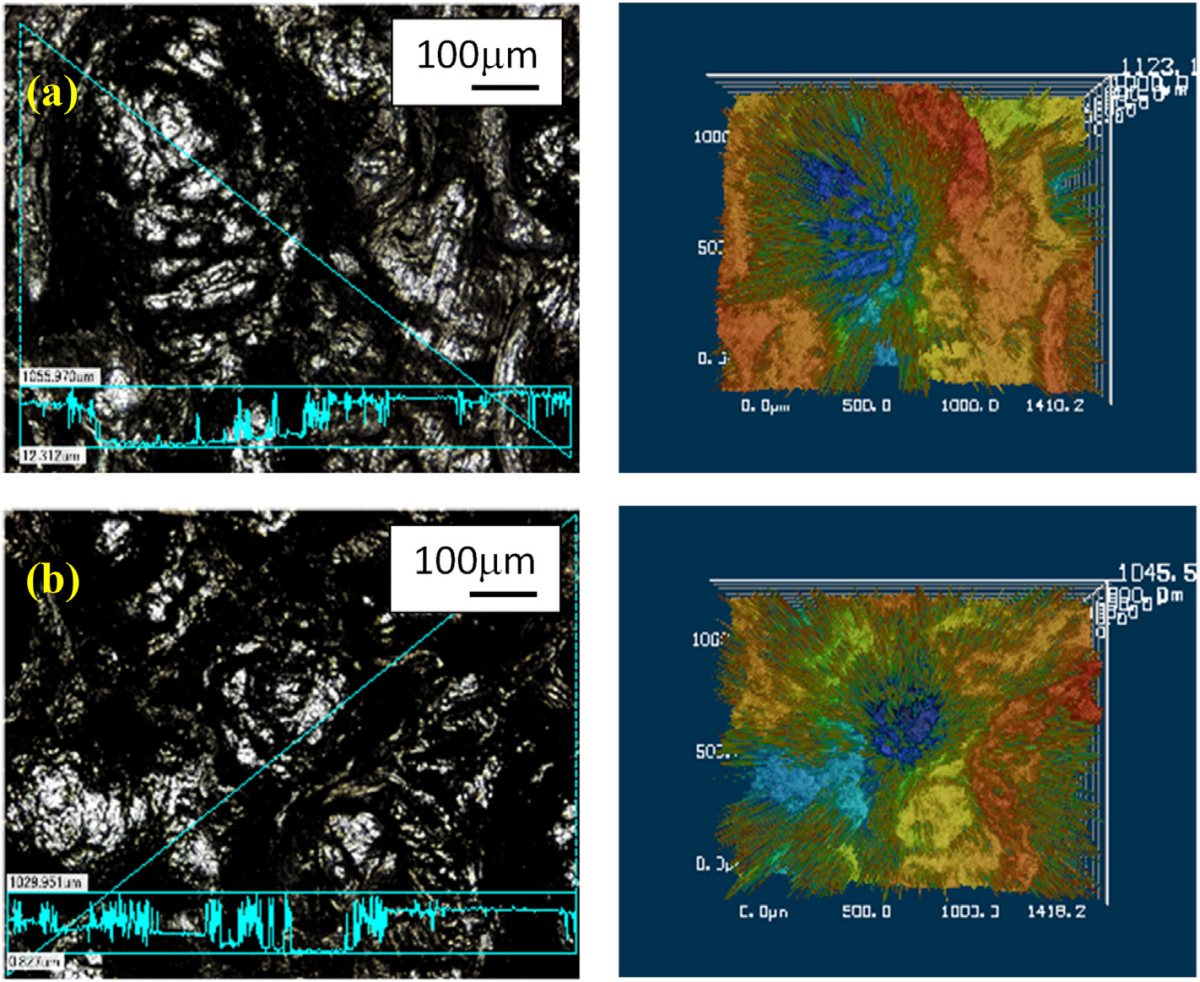
The roughness of pure aluminum specimens as determined using color laser microscopy (cutoff value: 8.0 mm) after (a) EI-MFC processing for 30 min (Ra: 300.48 μm) and (b) UTPC processing for 30 min (Ra: 217.53 μm).

Figure 5 presents the results obtained by examining both sample surfaces with a color laser microscope (KEYENCE Co., Ltd., VK9700/VK9710SP221). The arithmetic surface roughness in the peening region of the sample treated using EI-MFC was Ra = 300.481 μm (cutoff wavelength: λc; 8.00 mm), whereas the value for the UTPC specimen was 217.526 μm. These data indicate that the EI-MFC technique was superior to the UTPC process. It should also be noted that installing the EI-MFC nozzle running mechanism developed in previous research (Ijiri et al., 2021b) instead of the fixed point processing unit used in the present work, running at a constant speed under computer control, would significantly expand the machining area.

**Figure 6:**
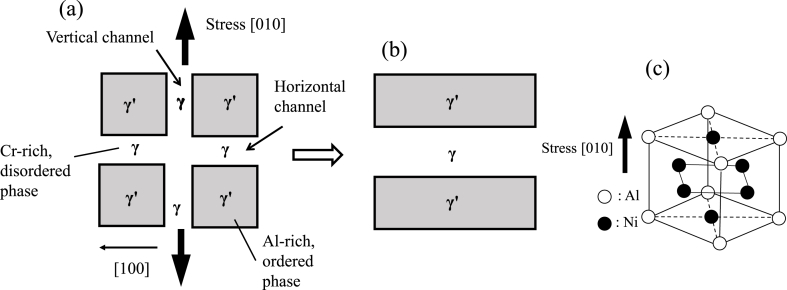
A diagram showing rafting behavior in a specimen (a) before rafting, (b) after rafting, and (c) L1_2_ structure.

**Figure 7:**
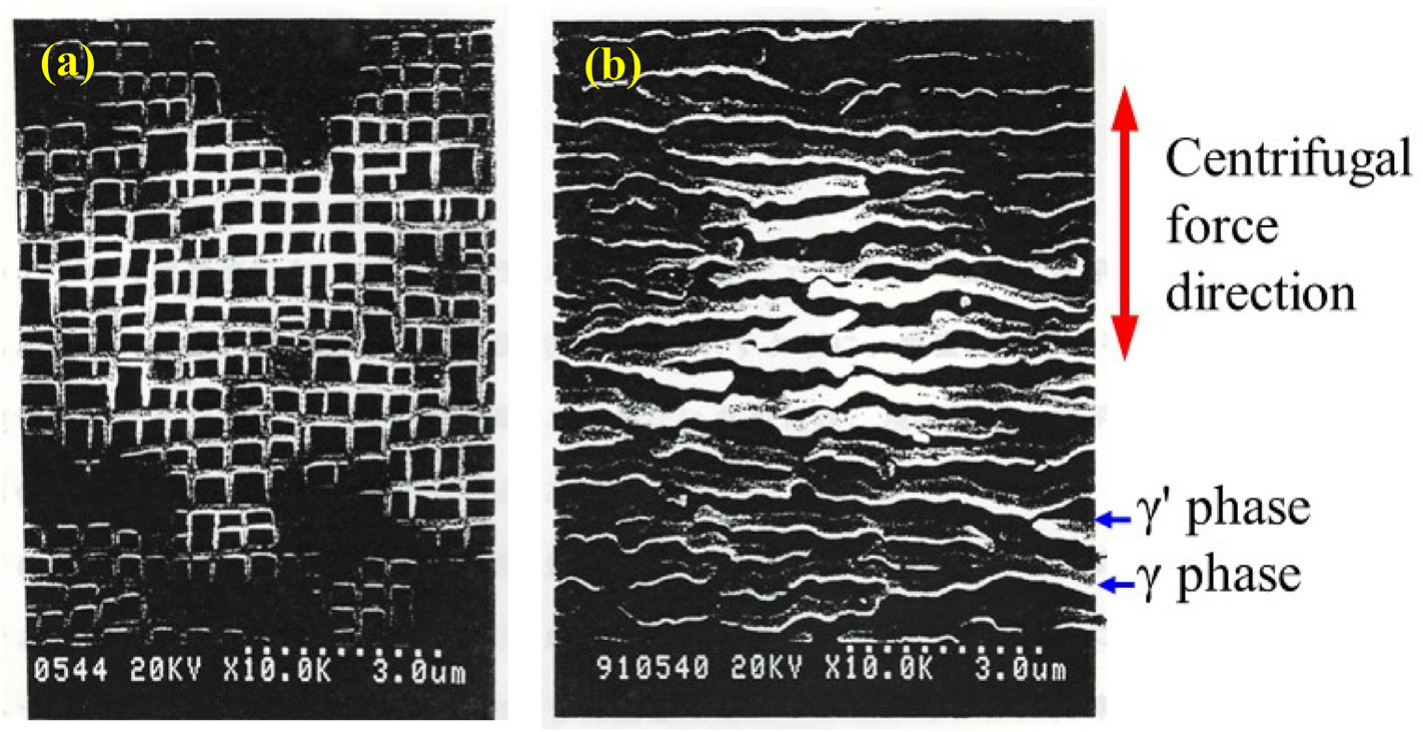
SEM images of a Ni-based superalloy sample (a) before rafting (that is, before creep deformation) and (b) after rafting (following creep deformation).

The Ni-based superalloys used in the rotor blades of gas turbines and jet engines have a two-phase structure consisting of lattice-shaped γ phases and cube-shaped γ′ phases (Figure 6). This grid-like two-phase structure in the precipitation strengthened material works to trap dislocations so that deformation of the metal is minimized. When manufacturing turbine blades, single-crystal, unidirectionally solidified columnar crystal Ni-based superalloys are precision cast so that the creep strength in the [010] direction is increased and the crystals are aligned in the [010] direction. However, above 1000 °C, the centrifugal force changes the structure of the metal such that the γ and γ′ phases are arranged perpendicular to the load direction (as shown in Figure 6). This creep damage is termed rafting, and can be limited by stabilizing the lattice structure through the addition of various elements. Figure 7 presents SEM images showing the microstructural changes in the γ and γ′ phases before and after creep damage. In the case that EI-MFC processing can form a structure (that is, induce horizontal rafting) in which the γ and γ′ phases are situated parallel to the load direction on the surface in the initial state of the metal, it is expected that the creep life will be extended.

Reed (2006) provided guidelines for controlling the microstructure of Ni-based superalloys, based on the following four categories of adjustments. (1) The concentrations of elements that stabilize the γ′ phase, such as Al, Ti and Ta, are tuned to adjust the volume fraction of the γ′ phase to about 70%. (2) The lattice mismatch between the γ and γ′ phases is minimized (known as microstructure stabilization). (3) The creep strength is improved by adding metals such as Re, W, Ta, Mo or Ru. However, because this also promotes the collapse of the two-phase structure, the amount that is added must be carefully selected. (4) Surface deterioration of the alloy at high temperature and in specific atmospheres is prevented by modifying the composition of the metal. It is possible that EI-MFC processing can be applied to realize items (1) and (4) in this list.

The mechanism of rafting has not yet been elucidated but γ′ rafting in response to complex stress has been studied experimentally and analytically by Caccuri et al. (2017). Tsukada et al. (2017) also used the phase field model (which permits direct calculation of the internal structures of materials on the mesoscale) to perform simulations related to rafting rate theory, and produced a crystal plasticity model based on mesoscopic dislocation density. Cottura et al. (2016) has reported that plasticity significantly slows the evolution of rafting based on a phase-field model combined with a density-based crystal plasticity model.

In the present work, the base material that was assessed was the Ni-based columnar crystal superalloy CM186LC/DC having the chemical composition summarized in Table 1.

**Table 1:** Chemical composition of the CM186LC/DS (mass%).

Cr	Co	Mo	Hf	Ta	W	Re	Ti	Al	Nb	C	B	Zr	Ni
6.0	9.3	0.5	1.4	3.4	8.5	3.0	0.7	5.7	-	0.07	0.015	0.07	Bal.

The CM186LC/DC specimen was solidified in one direction and then rapidly cooled after heating at 1080 °C for 4 h. Following this, the metal was again rapidly cooled after heating at 871 °C for 20 h as an aging treatment. This alloy contains 1.4% Re and exhibits the highest creep strength among current DS metals. In addition, it has the highest degree of corrosion resistance among the high-strength DS alloys (McColvin et al., 1997). Because the heat treatment applied to the present specimen after casting was only for the purpose of aging, and a solution treatment was not performed, the sample also had exceptional resistance to recrystallization.

After electrolytic polishing of the specimen, portions were subjected to either UTPC or EI-MFC processing and the mechanical metallographic changes in the γ and γ′ phases before and after processing were evaluated using several technique. Samples for analysis were prepared by cutting the metal using a wire-based electric discharge apparatus to obtain specimens with dimensions of 45 mm × 45 mm × 5 mm, having the (001) crystal plane on each sample surface. Each cut surface was subsequently finished with number 1500 emery paper and buffed. The microstructure of each specimen was exposed by immersing the metal in 10 ml hydrochloric acid combined with 90 ml ethanol for 25 s while applying a voltage of 3 V, after which scanning electron microscopy (SEM) was used to observe the microstructure. Line analyses for the various elements were also performed using SEM in conjunction with energy dispersive spectroscopy (EDS). The possibility that heat input to the metal surface during MFC or UTPC processing promoted the diffusion of elements between the γ′ and γ phases was examined. Changes in corrosion resistance before and after processing were investigated using Kelvin force probe microscopy (KFM). Variations in the coefficient of friction of each sample surface were assessed, employing lateral modulation friction force microscopy (LM-FFM). Prior to use, Ni-based superalloys that will be exposed to flame temperatures in excess of 1500 °C are covered with a thermal barrier coating (TBC) that reduces the temperature experienced by the metal by approximately 200 °C. The increased coefficient of friction resulting from UTPC or EI-MFC processing is expected to increase the bond strength of such TBC films to the metal.

In the present work, torsional vibrations of the untreated materials were investigated at three points randomly selected on the specimen surfaces, separated from one another by 5 μm. Specimens subjected to peening via cavitation were also examined in the same manner. Larger torsional angles of the LM-FFM cantilever are associated with higher coefficients of friction, and this technique generates potential values that reflect the magnitude of the coefficient. As discussed above, columnar crystals of a Ni-based superalloy will experience a strong centrifugal force at a high temperature of 1500 °C that leads to creep-related damage known as rafting. This phenomenon can induce a change in the material such that the γ′ and γ phases are vertically aligned in the centrifugal force direction of [010], while dislocations accumulate at the interfaces between the two phases. These accumulated dislocations can generate voids or cracks, leading to creep rupture. In this study, we investigated the possibility that either UTPC or EI-MFC processing could induce rafting-like phenomena on the metal surface.

Variations in the phase structure after cavitation processing were examined based on SEM observations at specific (x,y) coordinate points. Specifically, two disc-shaped specimen ends were scratched as markers, and peening was performed in the middle of these two markers.

## 3 Results and discussion

**Figure 8:**
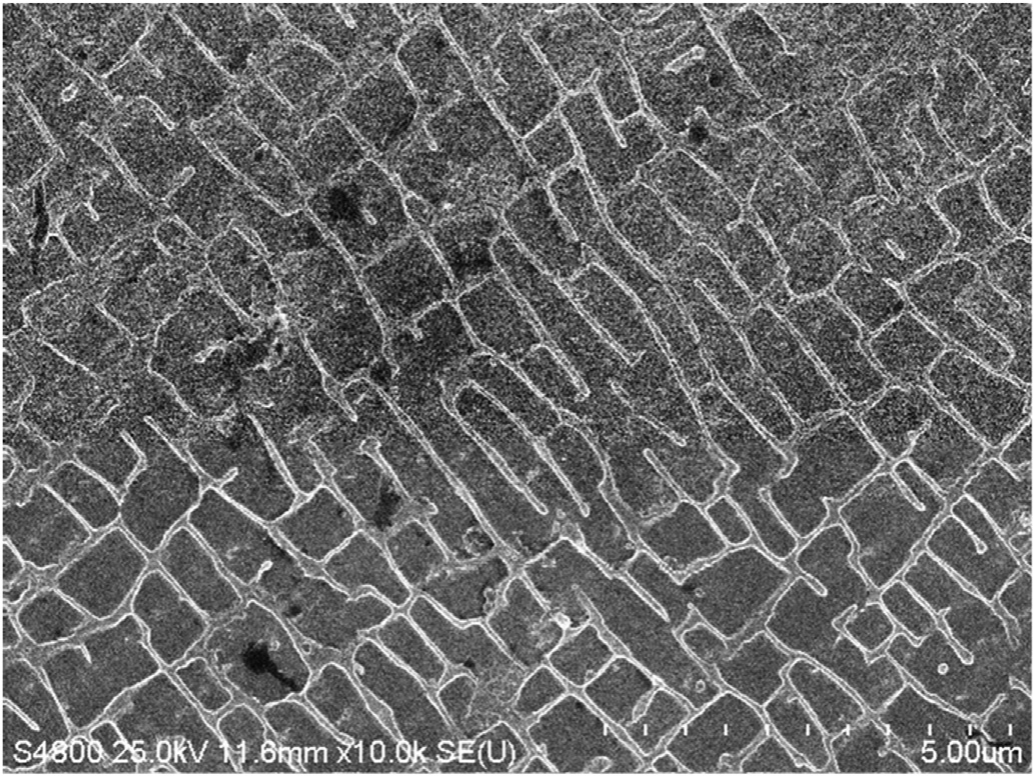
An SEM image of the surface microstructure before cavitation processing and after electropolishing.

The electropolishing conditions described in the experimental section clearly generated a lattice-shaped γ phase and a cubic-shaped γ′ phase, as shown by the SEM image of the CM186LC after electrolytic polishing in Figure 8. It is also evident that the dice-like shape of the γ′ phase and the grid-like shape of the γ-phase were imperfect, with some local disturbances.

**Figure 9:**
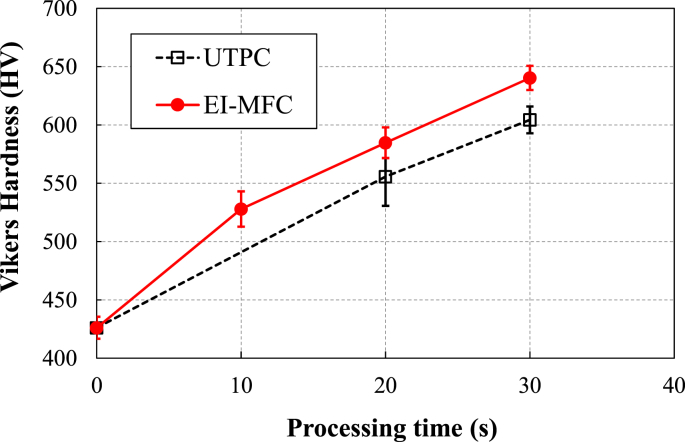
The relationships between processing time and micro Vickers hardness values during both UTPC and EI-MFC.

Figure 9 plots the micro Vickers hardness data as functions of processing time. The untreated material had a value of 426.2 HV, and both UTPC and EI-MFC treatments are seen to have hardened the metal as the processing time was increased. In addition, the hardness of the EI-MFC specimen exceeded that of the UTPC sample by 30 and 40 HV after 20 and 30 min, respectively. This difference is attributed to the more concentrated energy of the cavitation cloud during the EI-MFC process, meaning that the pressure and temperature in the cavitation bubbles were higher, with a greater degree of processing.

**Figure 10:**
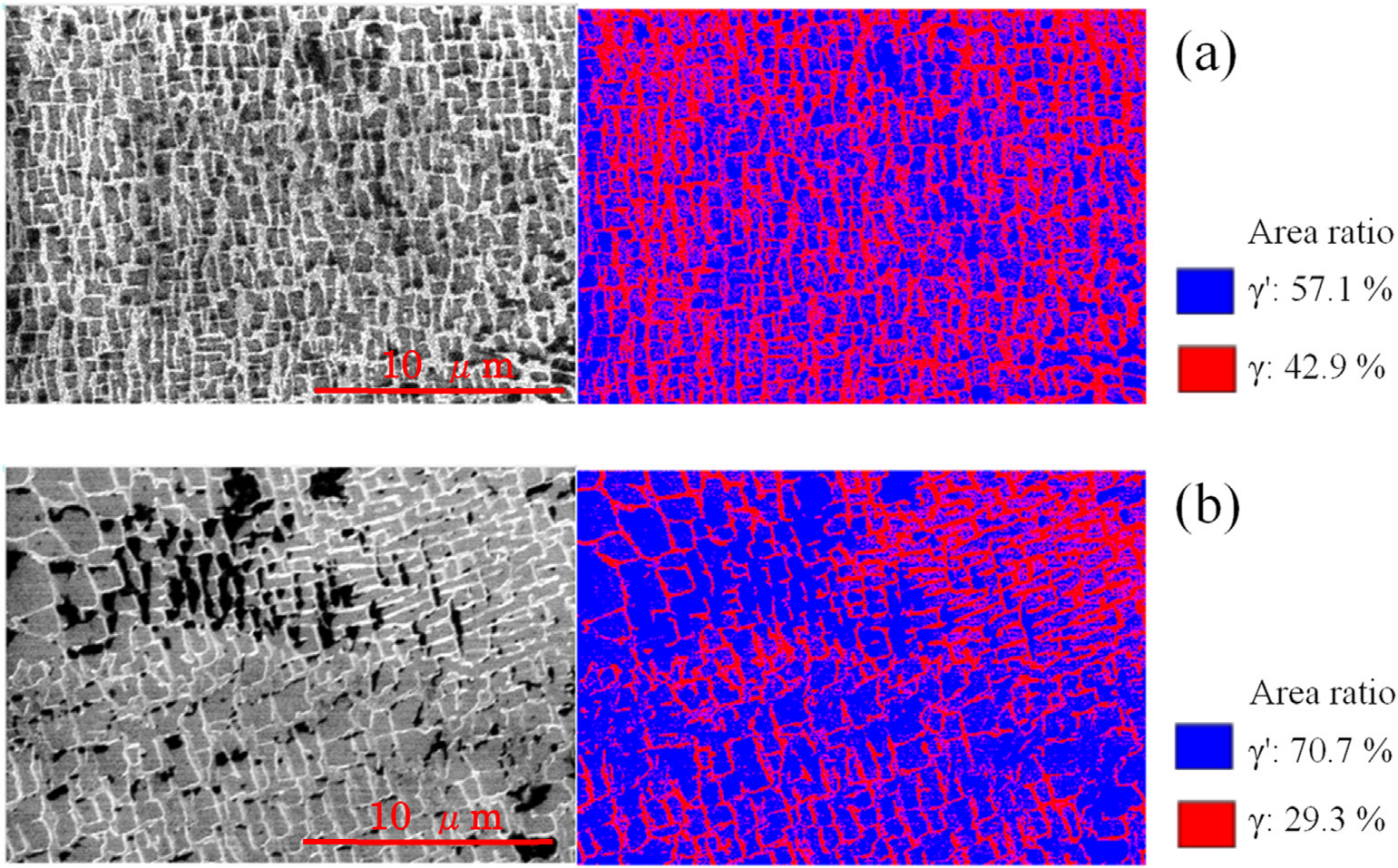
SEM and binarized images showing the γ′ phase proportions in a sample (a) before cavitation processing and after electropolishing and (b) after UTPC processing for 30 min.

Figure 10a shows an SEM image of a sample after electrolytic polishing and before UTPC processing, together with an image obtained by binarizing the SEM output. The area-based proportion of the γ′ phase in this material was 57.1%, and this value increased to 70.7% after UTPC processing (Figure 10b). This change resulted from deformation of the metal due to the pressure imparted by the ultra-high-temperature and high-pressure cavitation, and atomic diffusion due to the rise in surface temperature. If similarly enlarged γ′ phases are alternately laminated with grid-like γ phases just below the surface, the area fraction volume fraction of the γ′ phases will coincide with the volume fraction of the surface γ′ phase. This fraction was also about 70%, suggesting that the surface was modified so as to improve its creep resistance. As described above, it is possible to adjust the volume fraction of the γ′ phase, which has a higher creep resistance, to 70% by UTPC processing.

**Figure 11:**
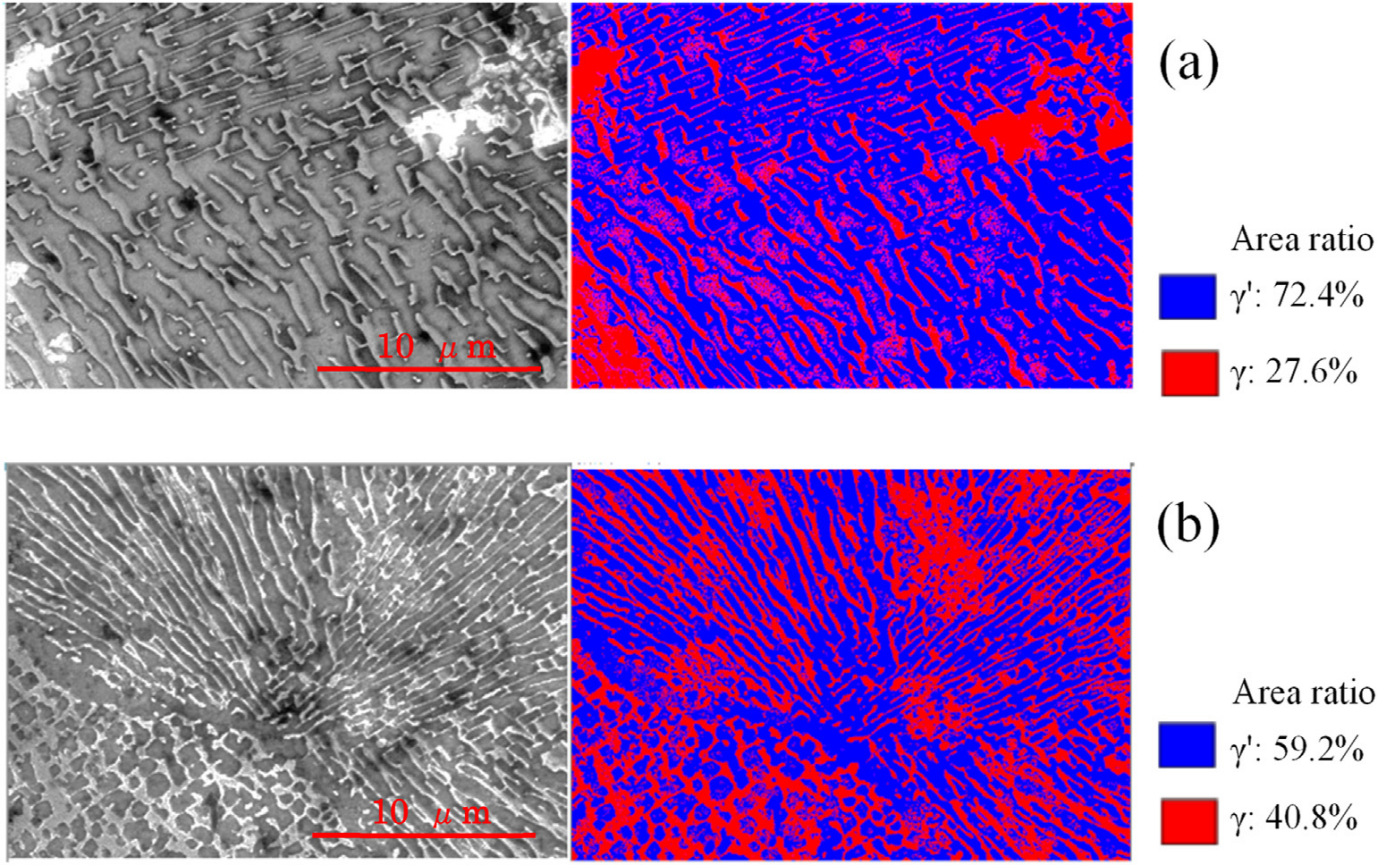
SEM and binarized images showing the γ′ phase proportions in a sample after EI-MFC processing for (a) 20 and (b) 30 min.

The images in Figure 10 demonstrate that, although the γ′ phase was expanded by UTPC processing, rafting did not occur. In contrast, during EI-MFC processing, rafting was found to proceed on the sample surface. An SEM image and binarized data for the sample after EI-MFC processing for 20 min are provided in Figure 11a, while Figure 11b shows the results after a 30 min EI-MFC treatment. After 20 min of processing, the γ phase was divided and began to align along one specific direction. Following 30 min, the divided γ phases were connected to produce rafting. Thus, we were able to reproduce in water the structural changes that are known to occur in a Ni-based superalloy heated to approximately 1300 °C. It is therefore likely that EI-MFC processing of the surface of a precision cast rotor blade would extend the creep life of the article by inducing horizontal rafting parallel to the centrifugal force direction on the surface.

**Figure 12:**
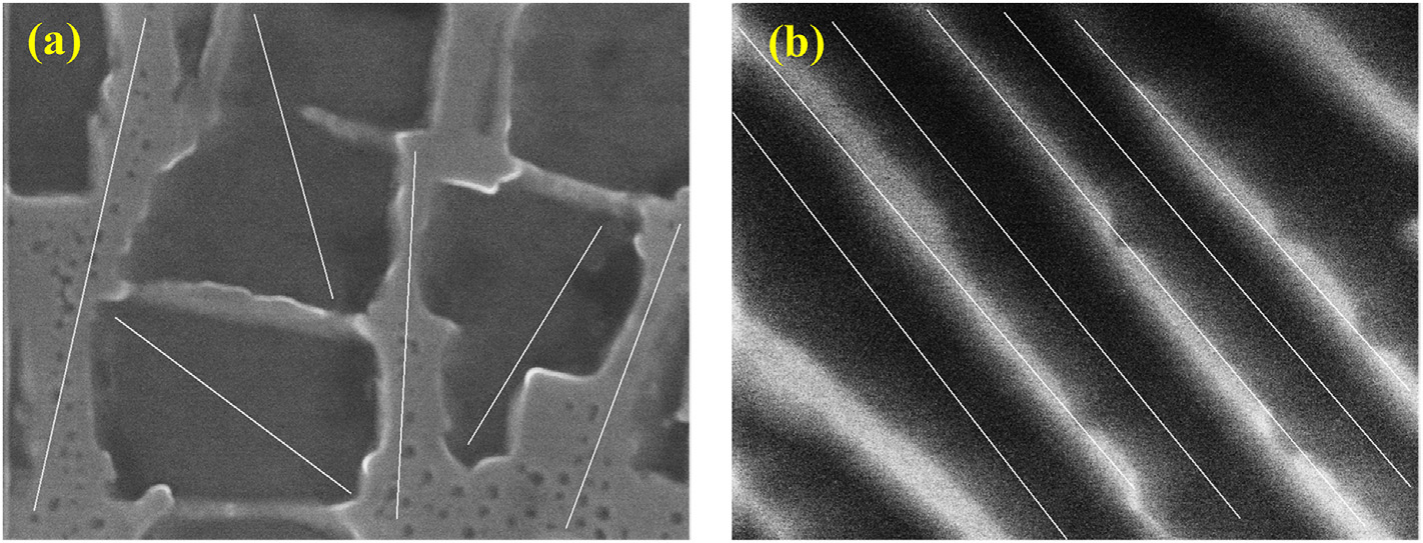
Examples of the SEM-EDS line analysis position for Al and Cr concentrations in samples processed using (a) UTPC for 30 min and (b) EI-MFC for 30 min.

Figure 12 and Table 2 presents the results of SEM-EDS line analyses of specimens treated using UTPC for 30 min (and having an expanded γ′ phase) and treated using EI-MFC for 30 min (in which rafting occurred). The values shown in this figure represent the average concentrations obtained from line analyses within ten regions of both specimens. In the case of the untreated material (after electrolytic polishing) and of the specimen subjected to UTPC for 30 min, the γ phase was Cr-rich while the γ′ phase was Al-rich, whereas the EI-MFC specimen had an Al-rich γ′ phase. It is known that atomic diffusion occurred between the γ and γ′ phases of a Ni-based superalloy single crystal during creep. In the present study, atomic diffusion was also promoted by EI-MFC processing.

**Table 2:** The average SEM-EDS line analysis results for Al and Cr concentrations in samples processed using (a) UTPC for 30 min and (b) EI-MFC for 30 min.

		γ phase	γ′ phase		γ phase	γ′ phase
Electropolishing		3.87	4.44		5.85	4.70
UTPC 30 min	Al (wt%)	3.85	4.42	Cr (wt%)	5.48	4.49
EI-MFC 30 min		4.47	3.75		4.58	4.33

**Figure 13:**
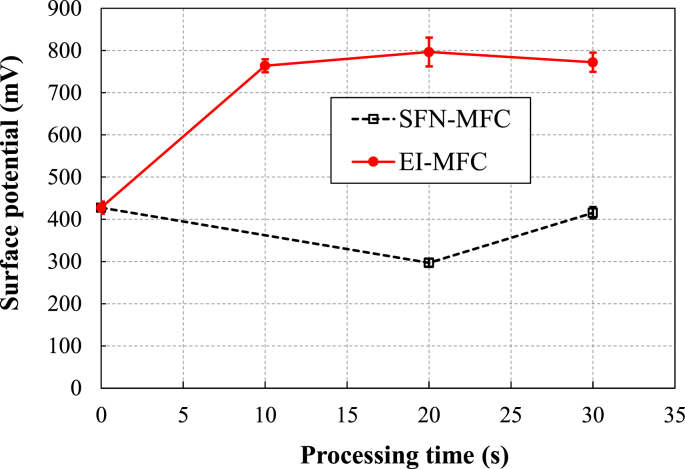
Surface potential obtained by KFM for UTPC materials and EI-MFC materials as functions of processing time.

Figure 13 shows the relationships between processing time and the surface potential for the UTPC and EI-MFC test pieces as measured by KFM. The surface potential (or corrosion potential) data were obtained by measuring four areas for each processing condition, performing three line analyses in each area, and taking the average of the total of 12 line analyses. These results demonstrate that the corrosion potential of the UTPC specimen was not changed after processing, while that of the metal subjected to EI-MFC was increased, meaning that the corrosion resistance was improved.

**Figure 14:**
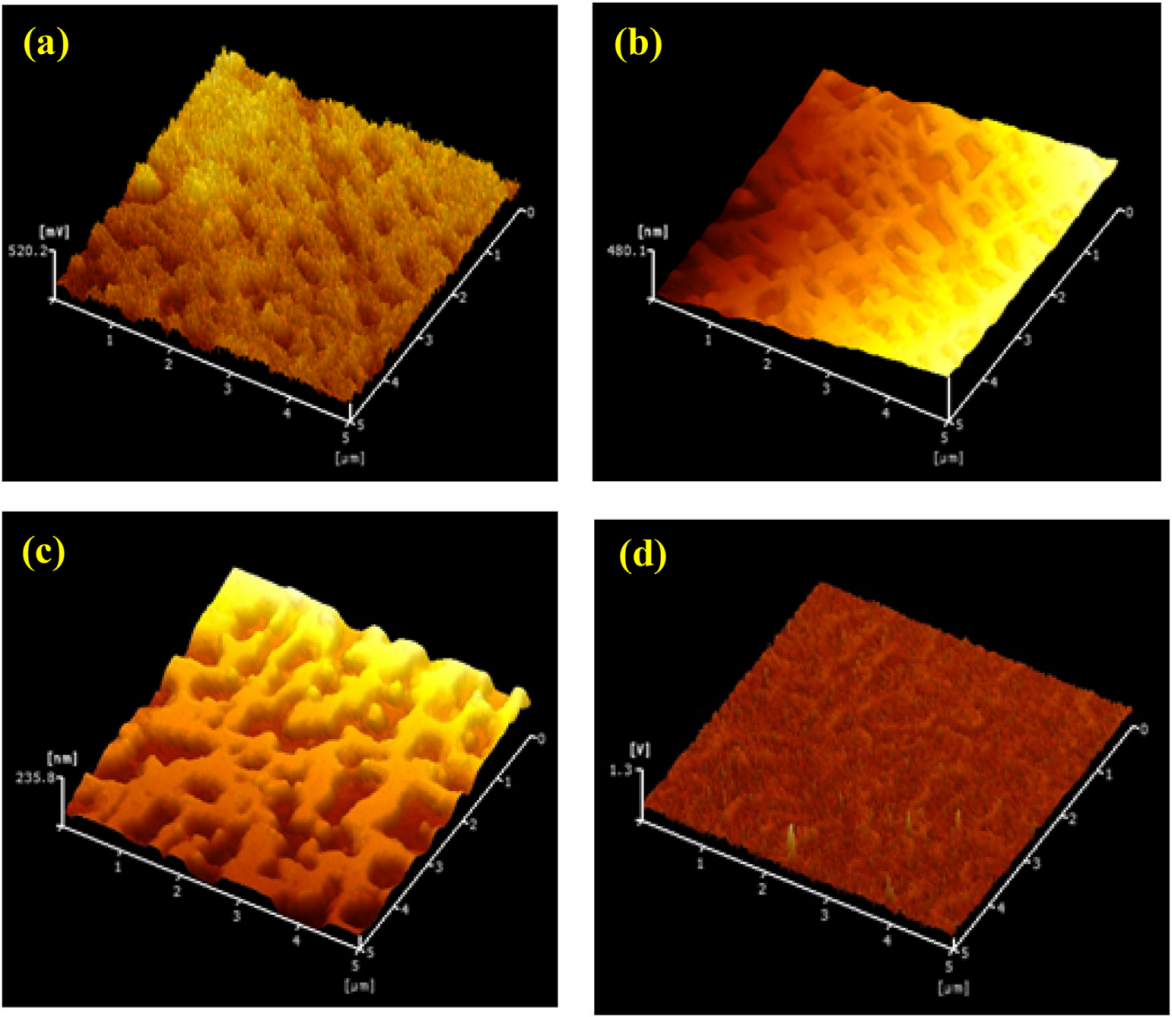
KFM results showing (a) a shape image and (b) corrosion potential for a sample processed by UTPC for 30 min, and (c) a shape image and (d) corrosion potential for a sample processed by EI-MFC for 30 min.

Figure 14 presents images showing the shapes and corrosion potential variations for the test pieces processed by UTPC and EI-MFC for 30 min, acquired using KFM. The shape images indicate the same grid-like γ phases and dice-like γ′ phases observed by SEM. The corrosion potential for the γ phase was evidently higher than that for the γ′ phase because the Cr concentration in the former was higher.

**Figure 15:**
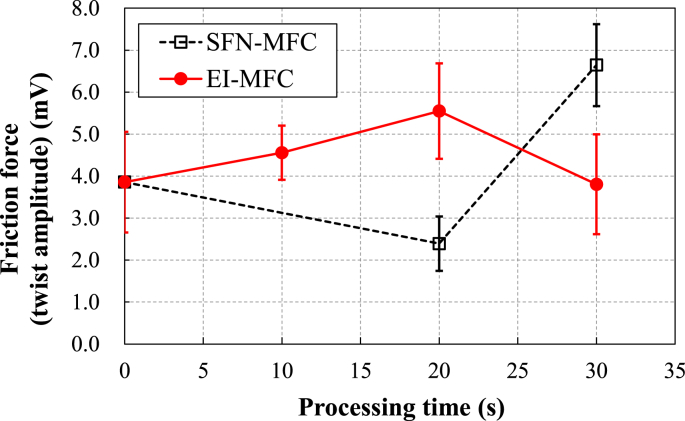
Friction force values obtained using LM-FFM for UTPC and EI-MFC materials as functions of processing time.

Figure 15 summarizes the relationships between processing time and the frictional force for the UTPC and EI-MFC specimens as determined using LM-FFM. These data were acquired by averaging a total of nine line analyses based on three line analyses in each of three areas on each specimen. This technique represents a type of SPM (Scanning Probe Microscope) measurement mode that assesses the frictional force distribution during atomic force microscopy. During this process, the sample is laterally vibrated while the torsional vibrations of the cantilever are imaged and variations in frictional force are generated. As a consequence, the instrument output is the potential in units of mV. This is an effective means of investigating differences in materials that cannot be ascertained from the shape and the distribution state of the metals. Because the effect of twisting of the cantilever due to unevenness is minimal, the friction characteristics of the materials can be studied.

**Figure 16:**
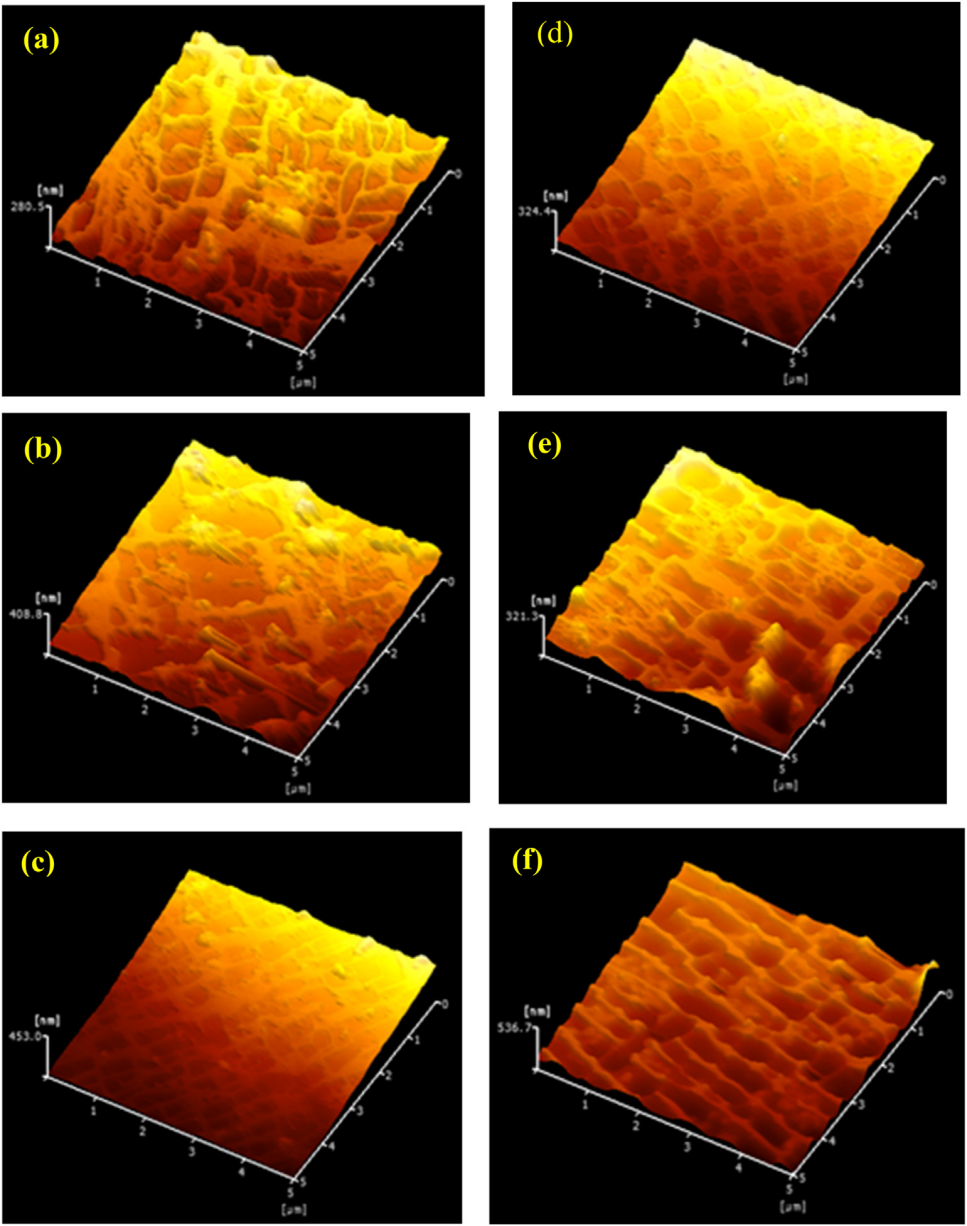
Shape images determined using LM-FFM for materials (a) before cavitation processing and after electropolishing, and after UTPC processing for (b) 20 and (c) 30 min or EI-MFC processing for (d) 10, (e) 20 and (f) 30 min.

Shape images acquired from the samples using LM-FEM are presented in Figure 16. As was evident in Figure 15, the average frictional force of the EI-MFC specimen increased along with the processing time, although the frictional force of the UTPC sample after 30 min was higher than that of the specimen treated using EI-MFC for 30 min. These results are attributed to alignment of the γ and γ′ phases in the scanning direction of the probe, as shown in the shape image in Figure 16f, which indicates that rafting occurred.

The increased coefficient of friction obtained from the EI-MFC processing suggests that a TBC (which will have a different coefficient of thermal expansion from the underlying metal) would become more difficult to remove. Thus, even if tensile or compressive thermal stresses were applied to the film/metal interface, the coating would remain intact. In the future, it would be helpful to repeat these tests while applying an actual TBC to a specimen after EI-MFC processing to confirm the improvement in peel strength.

The technique demonstrated herein is also expected to be applicable to the fabrication of hydrogen combustion turbines (which operate at 1700 °C with higher combustion efficiency than the current 1500 °C class gas turbines). The high processing energy that can be obtained using the EI-MFC technique could also increase the surface strength and surface flattening of Ni-based superalloys produced by 3D printers.

## 4 Conclusions

This work investigated the mechanism by which EI-MFC processing improves upon the UTPC method developed in previous research. Specifically, the surface strength, the coefficient of friction of the metal surface and the corrosion resistance of a CM186LC/DS specimen were all found to be enhanced following EI-MFC processing.

(1) EI-MFC processing gives better results within the peening area compared with conventional UTPC.
(2) When EI-MFC processing is performed, the surface hardness increases significantly with processing time and the final hardness value exceeds that obtainable from UTPC processing.
(3) The application of either UTPC or EI-MFC for the optimal time span improves the coefficient of friction for the metal surface.
(4) UTPC processing does not improve corrosion resistance, while EI-MFC processing does increase corrosion resistance.
(5) Prolonged EI-MFC processing of a CM186LC/DS specimen induces the same rafting phenomenon (that is, creep damage) that occurs upon heating at 1300 °C.

## Declarations

### Author contribution statement

Toshihiko Yoshimura: conceived and designed the experiments; analyzed and interpreted the data; wrote the paper.

Yuji Sugae, Masataka Ijiri: performed the experiments; analyzed and interpreted the data.

Takayuki Ogi, Fumihiro Kato: performed the experiments.

### Funding statement

This work was supported by the 10.13039/501100001691Japan Society for the Promotion of Science, KAKENHI Grants 19K04110 (Grant-in-Aid for Scientific Research C).

### Data availability statement

Data included in article/supplementary material/referenced in article.

### Declaration of interests statement

The authors declare no conflict of interest.

### Additional information

No additional information is available for this paper.

## References

[bib1] Caccuri V., Cormier J., Desmorat R. (2017). γ' rafting mechanisms under complex mechanical stress state in Ni-based single crystalline superalloys. Mater. Des..

[bib2] Cottura M., Appolaire B., Finel A., Bouar Y.L. (2016). Coupling the phase field method for diffusive transformations with dislocation density-based crystal plasticity: application to Ni-based superalloys. J. Mech. Phys. Solid..

[bib3] Gao Y.K., Wu X.R. (2011). Experimental investigation and fatigue life prediction for 7475-T7351 aluminum alloy with and without shot peening-induced residual stresses. Acta Mater..

[bib4] Hashish M. (1984). A modeling study of metal cutting with abrasive waterjets. J. Eng. Mater. Technol..

[bib5] Hashish M. (1991). Characteristics of surfaces machined with abrasive waterjets. J. Eng. Mater. Technol..

[bib6] Ijiri M., Yamaguchi K., Kikuchi S., Kato F., Kunieda Y., Sakurai H., Ogi T., Yoshimura T. (2021). Formation of a phosphoric acid compound film on an AZ31 magnesium alloy surface using cavitation bubbles. Surface. Interfac..

[bib7] Ijiri M., Yamaguchi K., Kikuchi S., Fujiwara M., Nakanishi Y., Yoshimura T. (2021). Improvement in the quality of the processed material surfaces lies in the moving of nozzle in the cavitation processing. Surface. Interfac..

[bib8] Kim Y.J., Bae D.H., Kim Y.J. (2005). The improvement of high cycle fatigue properties of AC4CH alloy with shot peening treatment. Key Eng. Mater..

[bib9] Kling C.L. (1970).

[bib10] McColvin G.M., Sutton S., Whitehurst M., Fleck D.G., Vranken T.S.V., Harris K., Erickson G.L., Jacqueline B., Wahl J.B. (1997). Proc. Of the Institute of Materials, Fourth International Charles Parsons Turbine Conference, Advances in Turbine Materials, Design and Manufacturing.

[bib11] Peng G., Tryggvason G., Shimizu S. (2015). Two-dimensional direct numerical simulation of bubble cloud cavitation by front-tracking method. IOP Conf. Ser. Mater. Sci. Eng..

[bib12] Peng G., Oguma Y., Shimizu S. (2018). Numerical simulation of unsteady cavitating jet by a compressible bubbly mixture flow method. IOP Conf. Ser. Earth Environ. Sci..

[bib13] Plesset M.W. (1949). The dynamics of cavitation bubbles. J. Appl. Mech..

[bib14] Pollock T.M., Tin S. (2006). Nickel-based superalloys for advanced turbine engines: chemistry, microstructure and properties. J. Propul. Power.

[bib15] Rayleigh L. (1917). On the pressure developed in a liquid during the collapse of a spherical cavity. Phil. Mag..

[bib16] Reed R.C. (2006).

[bib17] Saitou N., Enomoto K., Kurosawa K., Morinaka R., Hayashi E., Ishikawa T., Yoshimura T. (2003). Development of water jet peening technique for reactor internal components of nuclear power plant. J. Jet Flow Eng..

[bib18] Summers D.A. (1995).

[bib19] Summers D.A., Tyler L.J., Blaine J., Fossey R.D. (1987). Proc. of the Fourth U.S. Water Jet Conference.

[bib20] Tsukada Y., Koyama T., Kubota F., Murata Y., Kondo Y. (2017). Phase-field simulation of rafting kinetics in a nickel-based single crystal superalloy. Intermetallics.

[bib21] Yoshimura T., Shiraishi K., Takeshima T., Komura M., Iyoda T. (2014). Nano-level surface processing of fine particles by cavitation to improve the photocatalytic properties of titanium oxide. Nanosci. Nanotechnol. - Asia.

[bib22] Yoshimura T., Tanaka K., Yoshinaga N. (2016). Proc. of the 23rd International Conference on Water Jetting.

[bib23] Yoshimura T., Tanaka K., Ijiri M. (2018).

[bib24] Yoshimura T., Shimonishi D., Hashimoto D., Nishijima N., Ijiri M. (2021). Effect of processing degree and nozzle diameter on multifunction cavitation. Surf. Eng. Appl. Electrochem..

